# Promises and Challenges of MicroRNA-based Treatment of Multiple Myeloma

**DOI:** 10.2174/156800912802429355

**Published:** 2012-09

**Authors:** P Tagliaferri, M Rossi, MT Di Martino, N Amodio, E Leone, A Gullà, A Neri, P Tassone

**Affiliations:** 1 Medical Oncology, Department of Experimental and Clinical Medicine, Magna Graecia University and T. Campanella Cancer Center, Salvatore Venuta Campus, Catanzaro, Italy; 2 Temple University’s College of Science & Technology, Philadelphia, PA, USA; 3 Department of Clinical Sciences and Community Health University of Milan, Fondazione Cà Granda IRCCS Policlinico, Milano, Italy

**Keywords:** Experimental therapeutics, microRNA, miRNAs, multiple myeloma, nanotechnology, nucleic acid delivery.

## Abstract

MicroRNAs (miRNAs) recently emerged with a key role in multiple myeloma (MM) pathophysiology and are considered important regulators of MM cell growth and survival. Since miRNAs can act either as oncogenes or tumour suppressors, the potential of targeting the miRNA network arises as a novel therapeutic approach for human cancer. Potential strategies based on miRNA therapeutics basically rely on miRNA inhibition or miRNA replacement approaches and take benefit respectively from the use of antagomirs or synthetic miRNAs as well as from lipid-based nanoparticles which allow an efficient miRNA-delivery. The availability of experimental *in vivo *platforms which recapitulate the growth of MM cells within the specific human bone marrow microenvironment in immunocompromised mice (SCID*-hu *and SCID-*synth-hu*) provides powerful systems for development of miRNA-based therapeutics in MM. Preliminary findings on the anti-MM activity of synthetic miRNAs in such experimental models offer a proof-of-principle that miRNA therapeutics is a promising opportunity for this still incurable disease representing the rationale for a new venue of investigation in this specific field.

## INTRODUCTION

For many years, the standard treatment of multiple myeloma (MM) has been essentially based on various combinations of alkylating agents, anthracyclines and steroids with or without high dose chemotherapy supported by autologous stem cell transplantation. However, in the last decade the understanding of the MM pathophysiology has allowed the identification of relevant epigenetic or genetic events and the definition of a key role exerted by the non-tumour compartment of the bone marrow (BM) in the support of the growth and survival of MM cells. This information has proven fundamental for the development of new drugs and has allowed the translation of preclinical findings in clinical practice. Novel agents, such as bortezomib, thalidomide or lenalidomide have dramatically increased the armamentarium of anti-MM drugs and have led to improved response rate and increased survival of MM patients. However, despite these enormous advances and the availability of novel therapeutics, MM still evolves into a drug resistant phase and most patients die for progressive disease. Therefore, there is an urgent need of novel therapeutic strategies.

Recent important findings have allowed a deeper understanding of the molecular networks involved in the regulation of MM growth and survival [1, 2]. Moreover, novel evidence in the emerging field of non-coding RNAs is expected to produce ground-breaking advancements for the treatment of the disease. Among non-coding RNAs, microRNAs (miRNAs) play a critical role in the post-transcriptional regulation of gene expression [1, 3, 4] and may cause repression of protein synthesis or mRNA degradation. miRNAs may act as oncogenes (Onco-miRNAs) or tumour-suppressors (TS-miRNAs) [5, 6] and are significantly involved in key events of carcinogenesis [7, 8]. On these bases, miRNAs have recently elicited a growing interest as new potential tumour cell targets/anticancer agents due to their ability to target multiple genes involved in cancer promotion or repression [9]. Several miRNAs have been found to be deregulated in MM [1]. Although their involvement in the pathogenesis of MM has been clearly demonstrated, miRNA-based therapy is still in its infancy and few reports suggest potential clinical applications of miRNAs in MM therapy [10, 11]. The main challenge for an effective miRNA-based therapy still includes the effective delivery of the appropriate miRNA to the bone marrow microenvironment (BMM) and its uptake by malignant plasma cells in the absence of off-target effects [9]. In this light, the present review reports and discusses the potential anti-tumour activity of miRNAs and their promise as innovative strategies in the next future.

## BRIEF OVERVIEW ON miRNA NETWORK 

miRNAs are short RNA molecules (18-24 nucleotides length) that gained scientific relevance since their widespread eukaryotic expression, their highly conserved evolution across different species and their tissue specific biology were revealed. These non-coding RNA molecules are post-transcriptional regulators of gene expression and play an important role in physiologic and pathologic conditions. Aside from the long-standing knowledge of the other areas of oncogenomics, the role of miRNA deregulation in cancer development has been recently established. 

The discovery of miRNAs goes back to 1993 in Lee, Feinbaum, and Ambros labs where it was found that the lin-4 gene, involved in the timing of the larval development of *Caenorhabditis elegans*, did not code for a protein but rather for a small RNA [12]. Approximately ten years later, the relevance and importance of miRNAs became clear, as well as the knowledge of how these molecules work and regulate their targets. 

miRNA sequences represent 1% of the genome of different species and they are arranged within the intronic region in the same orientation of the pre-mRNA, providing a convenient mechanism for the coordinated expression of both miRNA and protein products. miRNA coding sequences can be transcribed under the control of their own promoters [7], while others are clustered in the genome with a multi-cistronic-like expression pattern. The latter is the case of the mir-15a-16-1 cluster and miRNAs located inside or near Hox Clusters. mir-15a-16-1 cluster falls in the q14 region of human chromosome 13, which is recurrently deleted in chronic lymphocytic leukemia [13]. Hox clusters represent a family of transcription factor genes that play a crucial role in normal cell physiology, differentiation and carcinogenesis. Moreover, miRNA coding sequences are often located at fragile sites of the genome or in regions partially involved in loss of heterozygosity or amplification, or common breakpoint regions [8]. Regarding the miRNA transcription process, miRNAs residing in introns share regulatory elements and primary transcript with their pre-mRNA, while no primary transcript has been fully defined for the other miRNAs presumably transcribed from their own promoters. The expression pattern of miRNAs is widely different being tissue specific and related to developmental stages [14]. Indeed, expression array technologies have revealed that miRNA expression profile is distinct for each cell type according to the developmental stage, providing a mechanism to finely tune the transcriptome. Moreover, Iorio &amp; Croce [15] have recently demonstrated that alterations in miRNA expression profile are common in human cancer. It can be therefore argued that the study of miRNA profiles provides relevant information for the molecular characterization of solid and hematologic malignancies [16, 17]. It is still matter of debate whether the relative abundance of certain miRNAs in cells is related to strong transcriptional activity or to slow decay. Moreover, lower average levels may be either due to reduced expression in all cells or expression limited to few specific cells. However, it is widely recognized that miRNA regulation undergoes a dynamic and fine tuning. Based on important experimental findings, the Pandolfi’s group has recently proposed the “competing endogenous RNA” (ceRNA) hypothesis [18]. These authors propose that mRNAs, large non-coding RNAs and pseudogene transcripts containing miRNA Response Elements (MRE) are involved in a large regulatory network which is based on the competition for a limited number of miRNAs. mRNA transduction to protein synthesis is therefore dependent both on transcriptional activity and negative regulation based on miRNAs availability.

**Fig. (1):**
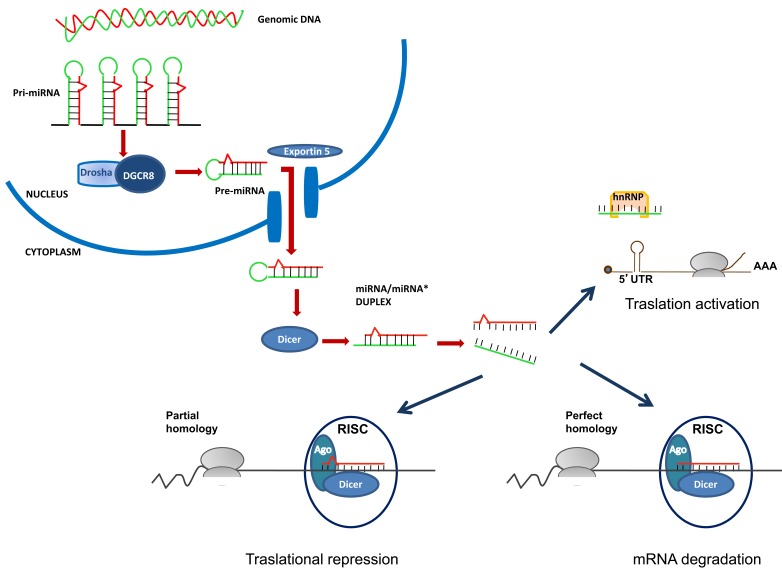
Schematic diagram of miRNA biogenesis and translational regulation. Mechanisms are described in the text. Abbreviations: DGCR8, Microprocessor complex subunit DGCR8, DiGeorge syndrome critical region 8; RISC, RNA-induced silencing complex; Ago, Argonaute; Drosha, RNA polymerase III family enzyme; Dicer, RNase III Dicer; hnRNP, heterogeneous nuclear ribonucleoproteins; 5’UTR, 5’ untranslated region; AAA, poly(A) tail.

miRNA is transcribed by a RNA polymerase II in a pri-miRNA hairpin that is cleaved by Drosha, a member of RNA polymerase III family, in a 70-100 bp pre-miRNA that translocates from the nucleus by exportin 5 (Fig. **1**). In the cytoplasm, Dicer, which also belongs to RNA polymerase III family, digests the pre-miRNA, producing a 20-22-bp miRNA/miRNA* duplex. One strand of the duplex is incorporated into the RNA-Induced Silencing Complex (RISC) that drives the mature miRNA strand to the 3’-UTR mRNA target sequence. 3’-UTR binding represses translation or induces deadenylation, the first step of the mRNA decay, depending on the degree of complementarity between miRNA and its target sequence. During the deadenylation process, the CAF1-CCR4 deadenylase complex is recruited following the interaction of the carboxy-terminal extremity of glycine-tryptophan 182 kD proteins (GW-182) with the poly(A) binding protein. The interaction of the GW-182 with Argonaute proteins (ARG), both core components of the miRISC complex, is essential for the miRNA repressive function [19-21]. Besides the canonical mRNA inhibition through the miRNA/3’UTR interaction, some miRNAs have been shown to bind to the open reading frame or to the 5’UTR of the target genes inducing activation of transcription rather than inhibition of protein synthesis [22]. Indeed, Eiring *et al.* have recently reported that miR-328 acts as a decoy factor by binding to a repressor ribonucleoprotein, independently from the seed sequence and RISC, and blocking it from translational repression of mRNA involved in myeloid cell differentiation [23, 24].

## miRNA DEREGULATION IN MM 

So far, several groups provided detailed analysis of miRNA expression patterns in MM. Based on the concept of a multistep pathogenesis of MM, evolving from MGUS, Pichiorri *et al.* [25] analyzed miRNA expression in different MM cell lines and in CD138^+^ primary PCs derived from healthy people and patients with either MGUS or medullary/extra medullary MM. They found that 48 miRNAs were significantly deregulated (up- or down-regulated) when comparing healthy plasma cells (PCs) and MGUS. If MM samples and healthy PCs were compared, the number of deregulated miRNAs raised to 74 (37 upregulated and 37 downregulated), suggesting that miRNA deregulation correlates with disease progression. Interestingly, the pattern of miRNA expression derived from MM cell lines was similar to that of MM patients mostly for upregulated miRNAs (90% of concordance) rather than downregulated ones (30% of concordance). Another study by Zhou *et al*. [26] compared miRNA expression profile of 52 newly diagnosed MM patients with that of 2 healthy donors. Among 464 human miRNAs analyzed, the authors found that mean expression levels of 95 miRNAs were higher in MM samples as compared to healthy donors. When performing a gene set enrichment analysis (GSEA) [27], higher total miRNA expression significantly correlated with expression of genes involved in cancer initiation and progression. Finally, an unsupervised hierarchical clustering divided the 95 expressed miRNAs in two differentially expressed clusters, where the cluster 1 patients had significantly higher gene expression profile-defined risk score as compared to cluster 2, suggesting a positive correlation between overall miRNA expression and risk scores [28]. An alternative approach to evaluate miRNA expression profile in MM has been adopted by Lionetti *et al*. [29]; in this study, patients (48 MM and 6 PCLs) were stratified according to translocation/cyclin (TC) classification [30], which recognized 5 distinct subgroups with distinct caryotipic abnormalities. Among 74 miRNAs, whose expression changed at least two folds from the mean across the dataset, 26 miRNAs were identified by multiclass analysis as highly differentially expressed across the 5 TC groups. These critical miRNAs were then stratified according to the type of genomic aberrations, namely 1q gain, 13 and 17 deletions and hyperdiploidy. The approach adopted in this study allows to correlate miRNA expression to main chromosomal alterations, that drive MM pathogenesis, thus relating miRNA pattern with gene expression profile and caryotipic signature. This attempt to integrate previous genomic data with the miRNA profile is likely to be preferred, although these studies cannot be completely comparable because of different microarray platforms used, including the total miRNA number analyzed, which is constantly updated. Overall these studies unveiled a number of miRNAs, which control critical genes in MM development. Some of these miRNAs will be further detailed in the next session.

### miR-15a and -16-1

These miRNAs are located on 13q14 chromosome, which is often deleted in MM patients. Roccaro *et al.* [10] found these miRNAs significantly downregulated in MM, as a consequence of chromosome 13 deletion. When transfected into MM cell lines, both miRNAs were able to inhibit proliferation and promote G1 arrest. Predicted targets of miR-15a and 16-1 include cyclins D1, D2, CDC25A, BCL2, PI3K, MAPK and interfere with NF-κB pathway activity. Overall, these data suggest a tumour suppressor role of both miRNAs in MM pathogenesis and provide a rationale for miRNA-based therapeutical approaches. 

### miRNA and p53 in MM

p53 mutation is a rare event in early stage MM while it occurs in patients with primary plasma cell leukemia (PPCL) or in MM patients who progress to a leukemic phase (secondary PCL, SPCL) [11]. Several miRNAs have been identified to regulate p53 expression and activity and/or are induced by p53. Pichiorri *et al.* [25] have shown that miR-181-a/-b, miR-106b~25 and miR-32 are up-regulated in MGUS, MM primary cells and cell lines. These miRNAs negatively modulate expression of p-300-CBP associated factor (PCAF). PCAF is a histone acetyl transferase which regulates transcription of several proteins, including p53. Suppression of miR-181-a/-b produced a significant delay in tumour development in a mouse model of MM, confirming that this miRNA nourishes MM tumour growth. Finally, miR-181-a/-b were significantly upregulated in two drug resistant MM cell lines when compared with parental line [31]. Pichiorri *et al. *[11] identified additional p53-induced miRNAs with an elegant approach: they selectively inhibited MDM2, the E3-ubiquitin ligase for p53, thus promoting p53 activation. miRNA changes upon p53 activation were then analyzed. Results showed that miRNA-34a, -192, -194 and -215, are upregulated and mediate p53 anti-proliferative activity in MM. Interestingly, miRNA 192, 194 and 215 are grouped in two clusters (192-2-194 and 194-1-215) located at 11q13.1 and 1q41.1, respectively. These are recognized as critical regions in MM pathogenesis. Indeed, these miRNAs target MDM2 and IGF-1, that control MM development and progression. The two clusters of miRNAs were found to be downregulated in MM, probably by promoter hyper-methylation as detected in MM cell lines. miRNA-34a methylation has been described in hematological malignancies, including NHL and MM [32]. 

### 17~92 Cluster 

According to Pichiorri *et al.* [25], this cluster is specifically upregulated in MM as compared to MGUS or normal PCs. Among others, cluster members include miR-19a, -19b, and miR-32. The role of miR-32 as indirect regulator of p53 has been already described above. miR-19a and -19b have been identified as negative regulator of SOCS-1, a protein that controls IL-6 mediated signaling. SOCS-1 downregulation induces constitutive STAT3 phosphorylation, which is reversed when MM cell lines are transfected with anti miR-19. Furthermore, miR-19 targeting downregulates the expression of BIM, a proapoptotic gene, that has been described to be expressed under the control of 17~92 cluster in other malignancies [33].

### mir-21 

This miRNA has been described as upregulated both in MM and MGUS as compared to normal PCs [25]. In MM, miR-21 is induced by IL-6 through STAT-3 signaling [34], suggesting that this miRNA works as survival and proliferative agent for malignant PCs and depends upon a critical micro-environment factor present in MM BM milieu. Moreover, miR-21, as well as miR-181-a/-b, is upregulated in two drug resistant MM cell lines when compared with parental line [31]. This feature is typical of end-stage MM and suggests a key role of miR-21 in MM progression. 

### miR-193b-365

Unno *et al.* [35] provided the first evidence of a new miRNA cluster, miR-193b-365, which is overexpressed in MM samples as compared to healthy PCs. The cluster is perturbed also in breast and ovarian cancers and appears to be highly conserved among vertebrates, thus confirming its relevance. However, to best of our knowledge, no data on potential role of this cluster in MM pathogenesis have been published to date.

### DICER and DROSHA in MM

miRNA levels can be influenced by activity of their master regulators, namely DICER and DROSHA complexes. In a recent report, Sarasquete *et al.* [36] evaluated Dicer/Drosha levels in MGUS, smoldering MM and symptomatic MM patients. Indeed, Dicer is upregulated in healthy PCs as well as MGUS PCs, while the expression is significantly lower in smoldering and symptomatic MM. Interestingly, Dicer expression significantly correlated with better PFS in symptomatic MM patients. In contrast, Drosha levels did not differ among MGUS and MM patients. Although these data need to be confirmed in larger cohorts of patients, these results resemble what has been observed in other studies in lung and ovarian cancers [37, 38]. 

## THE RATIONALE OF miRNA-BASED THERAPEUTIC STRATEGIES

miRNAs represent a basic network which regulates a variety of cellular functions. It is now becoming clear that several human diseases are indeed related to deregulated expression of miRNAs. It is therefore conceivable that the treatment of such diseases should rely on the reconstitution of underexpressed miRNAs or on the antagonism of aberrantly expressed miRNAs. miRNAs in human cancer represent indeed a paradigm in the general concept of miRNA therapeutics [39]. Such a paradigm, as clearly enunciated by Duchaine and Slacks [40], has recently led to the “double strategies” concept: miRNAs as anticancer agents/ miRNA as cancer targets. We will try to describe such approaches with perspectives and pitfalls in a rapidly developing field. 

### miRNA as Cancer Targets

The concept of “*miRNAs as a target*” approach needs to be discussed in the general scenario of targeted therapy. Although several molecular-targeted agents (MTAs) have indeed produced a novel paradigm for the treatment of human cancer, it has to be pointed that in many cases MTAs have produced modest benefits in long-term outcome of cancer patients. Major restrains of molecular targeted therapy are: *i*) the redundancy of signaling pathways in eukaryotic cells that impairs the capacity of single target inhibition to produce persistent tumour suppression, even in cases of demonstrated “target addiction” as for the activating mutations in the Epidermal Growth Factor receptor (EGFR) in non-small cell lung cancer (NSCLC), occurring in non smoking or light smoking individuals [41]; *ii*) the intrinsic complexity of cancer cells that make highly problematic the identification of the effective target in most malignancies [42]. Therefore, the shift from single drug /single protein targeting to a pattern-targeting strategy is a compelling issue. miRNAs have indeed the potential to target a number of different mRNAs in a coordinate fashion, thus producing more relevant perturbations in the tumour cell homeostasis.

Recent experimental findings have shown that tumours become addicted to oncogenic miRNAs. Medina *et al.* [43] have demonstrated that in mice conditioned to express miR-21, a pre-B malignant lymphoid disease arises, with these tumours being strictly dependent on miR-21 expression. Such experimental approach has provided a formal proof of miR-21 as an oncomir [43], substantiating the available data from molecular profiling studies and suggesting the existence of oncomirs [44]. A similar formal proof of oncomir evidence is available for miR-155 as reported by Costinean *et al.* [45]. All together, these findings provide the rationale for the design of anticancer strategies based on the inhibition of oncomirs by different approaches.

### miRNAs as Anticancer Agents

The alternative strategy can be defined “*miRNAs as an anticancer drug*”. This approach would rely on the opportunity of reprogramming tumour cells by delivery of a synthetic mature miRNA precursor. Many molecular profiling studies from ground-breaking work from Croce and Calin [13] have indeed demonstrated that a variety of miRNAs are indeed lacking in tumour cells and that their reconstituted expression leads to the reversal of the malignant phenotype [46]. The theoretical assumption of this approach is that miRNAs defective in tumour cells, that have demonstrated capacity to inhibit the expression of genes involved in tumour cell growth and survival, should be identified and replaced within the tumour tissue. On this regard, important information has been achieved by *in vivo* delivery of TS-miRNAs. Wiggins *et al.* [47] have recently demonstrated that synthetic miR-34a, complexed with a neutral lipid based delivery system, produces impressive regression of mice xenografted with SCLC cell lines either by direct intra-tumour injection or intravenous administration. This work has led to important observations. The formulated synthetic miR-34a: *i*) can be delivered in the tumour xenografts by direct intratumour or intravenous injection *ii*) does not produce toxicity in mice, *iii*) does not lead to cytokine release, and *iv*) is effective also against tumours which show normal levels of miR-34a. Therefore, such findings expand the concept of miRNA replacement. In order to be active, synthetic miRNAs are not required to reconstitute the levels of highly downregulated miRNAs. A possible explanation may be that synthetic miRNA sensitivity may be linked to target availability. All these points warrant further investigations.

Although current available data do not allow to predict toxicity of “*miRNAs as a target*” *versus* “*miRNAs as an anti-cancer drug*” approaches, some theoretical considerations can be made. In order to be effective the miRNA-targeting strategy needs indeed a driver oncogenic function of the targeted miRNA. As discussed above, such function has achieved a formal definition for miR-21 and miR-155, only. Moreover, off target effects in normal tissues are expected when inhibiting miRNAs that play a critical role in the homeostasis of cells, *e.g.* the stem cell compartments, as compared to replacement approaches which might lead to relative abundance of downregulated miRNAs inside the malignant cells for short term exposure. These points have been recently discussed [48]. 

## STRATEGIES AND CHALLENGES FOR *IN VIVO* miRNA DELIVERY

An important issue for nucleic acid-based therapeutic approach is the efficient delivery in tumour tissues and the uptake by tumour cells. Although miRNAs have been considered as intracellular network regulators so far, novel findings are clearly demonstrating that miRNAs play a substantial role as extracellular messengers and these latter findings are ready to open new avenues for efficient systemic delivery of therapeutic miRNAs [49]. The therapeutic delivery of nucleic acids remains a challenging issue and two non-viral basic strategies are being explored for delivery of therapeutic oligonucleotides: *i*) lipid-based delivery systems and *ii*) polymer-based carriers for oligonucleotide delivery.

Liposomes based on cationic lipids are the mostly investigated lipid-based delivery systems. The development of carrier-encapsulating oligonucleotides based on ionizable lipids as stabilized antisense lipid particles (SALPs) or stable nucleic acid lipid particles (SNALPs) has provided an important advancement in the field. Polymeric micro- and nanostructured platforms have been also widely investigated [50, 51]. An important and still open issue is the use of targeting molecules to be included in the delivery platforms like monoclonal antibodies or ligands for surface receptors (Ferritin). The development of delivery strategies is a major area of investigation for academic and corporate research institutions and it can be easily predicted that the efficacy of miRNA therapeutics will mostly rely on an efficient delivery system. Table **1** summarizes recent preclinical studies of miRNA treatment of human tumours [52-59].

## POTENTIAL miRNA-BASED STRATEGIES FOR MM

The basic rationale of targeting miRNA network has been based on studies where miRNA profile retains a prognostic role. Chi *et al.* have recently shown that miRNA expression profiles are correlated with genetic subtype and isotype. Moreover, ten miRNA appeared to be correlated with patient survival, providing evidence of their role in the clinical behavior of disease [60]. So far, while the biology and the expression features of miRNAs among several cancers has been significantly explored, only few reports in experimental systems support the notion that miRNAs may represent a therapeutic tool for MM. Roccaro *et al*. [10] demonstrated a down-regulation of 15a/16 in MM and, since these miRNAs appear to be negative regulators of cell proliferation, they suggested that the reconstitution of normal miRNA expression could represent a MM treatment. Pichiorri *et al. *[11] demonstrated miR-192, 194 and 215 downregulation in a subset of newly diagnosed MMs and on this basis suggested a new potential therapeutic strategy. More recently, Di Martino *et al.*^1^ first proved that either transient expression of miR-34a synthetic mimics or lentivirus-based stable enforced expression of miR-34a gene triggered growth inhibition and apoptosis in MM cells* in vitro*. Importantly the authors showed that synthetic miR-34a exerted also an *in vivo* anti-tumour activity in different clinically relevant models of human disease including in the SCID-*synth-hu *[61] model in which 3D biosynthetic scaffolds were reconstituted with human bone marrow stromal cells and then injected with human MM cells. Specifically this model allows to assess the activity of miRNAs against MM cells interacting with a human adult BMM. In all models, a significant tumour growth inhibition and/or survival improvement were observed without systemic toxicity. Importantly these authors achieved anti-tumour effects by a novel neutral lipid formulation of synthetic miR-34a supporting a rationale for development of miR-34a-based therapeutic strategies in MM. Interestingly, Stebner *et al.*^2^ suggested a positive interaction of miR-34a with bortezomib which underlines the possible relevance of this strategy in the treatment of MM. The authors detected low miR-34a levels in MM cells with epigenetic silencing of miR-34a and TP53 mutations while high miR-34a levels were detected in MM cells highly sensitive *in vitro* to bortezomib. To support a combined drug/miRNA replacement strategy, the authors demonstrated a more powerful growth impairment of xenografted lentivirus infected MM cells overexpressing miR-34a, if transduced cells were also exposed to bortezomib.

## SUMMARY

At present, it is possible to speculate that miRNA-based treatment of MM might indeed arise as a novel approach of the therapy of this important disease. In this light, there are in fact some points that need to be considered:

- miRNA profiling studies provided evidence of miRNA role in the pathophisiology of the disease;
- preliminary *in vitro* and *in vivo* study demonstrated that synthetic miRNAs exert antitumour activity and synergize with conventional drugs like bortezomib;
- effective nanotech delivery systems are presently available and are expected to be readily improved by the current research on this specific topic.

Taking in account the biologic complexity of MM, it can be predicted that an individualized approach will have high chance of providing an effective therapeutic tool. At this aim, it is conceivable that apart from the testing of candidate miRNAs on the basis of molecular profiling analysis of malignant plasma cells or even on circulating miRNA signatures, an alternative approach might be the use of screen platforms which might allow a wide search for novel candidate miRNAs for “miRNAs as a target” or “miRNAs as an anticancer drug” approaches [62]. The last point is the possible integration of miRNA therapeutics in the currently available treatment of MM. It is of course important to achieve novel information on miRNA activity but it is clear that miRNA therapeutics is a different approach as compared to molecular targeted agents in that it is a “pathway targeting approach” and that resistance might be a late event. miRNA therapeutics can therefore find place after conventional drug cyto-reductive strategies. In conclusion the track for synthetic miRNA/anti-miRNA development as investigational new drugs opens a new exciting scenario for the treatment of this important malignancy in the next future.

## Figures and Tables

**Table 1.** Recent preclinical studies of human tumours treated with miRNA.

Cancer	microRNA	Delivery	Dispensing Route	Reference
Breast carcinoma	miR-10b	2′-O-Me-modified	Tail vein	54
	duplex miR-22	RNA-jetPEI (polyethylenimine)	Local	55
Non-small cell lung cancer	Pre-miR-34a	Lipid-based delivery vehicle	Local and systemic	47
	Pre-miR-let7b	Lipid-based delivery vehicle	Local and systemic	52,53
Lung cancer	Pre-miR-133b	cationic lipoplexes	Systemic	56
Colon carcinoma	miR-145 and miR-33a	Low molecular weight PEI/miRNA complexes	Local and systemic	57
Prostate tumor	Pre- miR-16	Atelocollagen	Systemic	58
Hepatocarcinoma	miR-143	hsa-miR-143 gene expressing vector	Intratumoral	59

## Footnotes

1 Di Martino MT, Leone E, Amodio N, Foresta U, Lionetti M, Pitari MR, Gallo Cantafio ME, Gullà A, Morelli E, Leotta M, Tomaino V, Rossi M, Negrini M, Ferrarini M, Neri A, Anderson KC, Munshi NC, Tagliaferri P, and Tassone P. ASH meeting 2011, Abstract 2910.

2 Stebner E, Neri P, Johnson J, Gratton KJ, Ren L, Duggan P, Stewart DA, Bahlis N. Mir-34a sensitizes Multiple Myeloma (MM) Cells to the Proteasome Inhibitor Bortezomib. ASH meeting 2011, Abstract 652.

## ABBREVIATIONS

ARG: = Argonaute protein
CAF1-CCR4: = Deadenylase complex proteins
ceRNA: = Competing endogenous RNA
EGFR: = Epidermal Growth Factor receptor
GW-182: = Glycine-tryptophan 182 kD proteins
IGF-1: = Insulin-like growth factor-1
MGUS: = Monoclonal Gammopathy of Undetermined Significance
MM: = Multiple myeloma
MRE: = miRNA Response Elements
MTAs: = Molecular-targeted agents
NHL: = Non hodgkin lymphoma
NSCLC: = Non-small cell lung cancer
PCs: = Plasma cells
PEI: = Polyethylenimine
PFS: = Progression free survival
RISC: = RNA-Induced Silencing Complex
SALPs: = Stabilized antisense lipid particles
SCID-: = Severe Combined Immunodeficient-
SCID*-hu*: = Severe Combined Immunodeficient-human MM mouse model
SNALPs: = Stable nucleic acid lipid particles
*synth-hu*: = biosynthetic-human MM mouse model
3’-UTR: = 3’ untranslated region
5’-UTR: = 5’ untranslated region
